# Healthcare workers’ perspectives on HIV pre-exposure prophylaxis delivery in Sub-Saharan Africa: implications for injectable formulation rollout—a narrative review

**DOI:** 10.3389/fpubh.2026.1818059

**Published:** 2026-06-22

**Authors:** Nomvuselelo Nomzamo Mbatha, Nomakhosi Mpofana, Dumile Gumede

**Affiliations:** 1 Faculty of Health Sciences, Durban University of Technology, Durban, South Africa; 2 Department of Somatology, Faculty of Health Sciences, Durban University of Technology, Durban, South Africa

**Keywords:** cabotegravir, healthcare workers, HIV pre-exposure prophylaxis, implementation science, injectable PrEP, narrative review, oral PrEP, Sub-Saharan Africa

## Abstract

**Background:**

Long-acting injectable Human Immunodeficiency Virus (HIV) pre-exposure prophylaxis (PrEP), particularly cabotegravir, presents a promising HIV prevention option in Sub-Saharan Africa (SSA), where HIV prevalence remains high. As frontline providers, healthcare workers (HCWs) are central to PrEP delivery. Given the emerging introduction of injectable PrEP in SSA, this review examines HCW perspectives on PrEP delivery broadly, including both oral and injectable formulations, with particular attention to how oral PrEP implementation experiences inform injectable PrEP readiness.

**Methods:**

A narrative review was conducted with a comprehensive literature search across PubMed, Scopus, Web of Science, EBSCOhost, and Google Scholar for English-language studies published 2020–2025. Eligible studies comprised qualitative, quantitative, and mixed-methods primary research that examined healthcare workers’ knowledge, perceptions, attitudes, and readiness to deliver HIV PrEP in SSA. Relevant studies were identified, reviewed, and analyzed thematically to identify key patterns and implementation considerations.

**Results:**

A total of 742 records were identified across databases, and following review, 5 primary studies met the scope criteria, all examining oral PrEP implementation in Uganda, South Africa, Kenya, and Zimbabwe. Findings revealed varied HCW awareness and knowledge, often influenced by prior exposure to pilot initiatives. Attitudes were generally positive, emphasizing PrEP’s potential to enhance adherence and reduce stigma. Reported barriers included limited training, inadequate infrastructure, increased workload, and the absence of clinical guidelines. Key facilitators included task-shifting, government commitment, targeted capacity-building, and peer-led learning. These oral PrEP implementation lessons directly inform the readiness of injectable PrEP rollout.

**Conclusion:**

Healthcare workers in SSA display positive attitudes but uneven readiness to deliver PrEP. While most evidence derives from oral PrEP implementation, lessons learned on training needs, infrastructure requirements, task-shifting strategies, and health system readiness directly inform the introduction of injectable PrEP. Addressing gaps in training, guidelines, and resources, alongside strengthening policy and system support, is essential for the effective integration and scale-up of both oral and injectable PrEP formulations.

## 1 Introduction

Human Immunodeficiency Virus (HIV) pre-exposure prophylaxis (PrEP), a biomedical prevention strategy in which HIV-negative individuals take antiretroviral medicines to significantly reduce their risk of HIV acquisition, was formally introduced into public sector healthcare systems in Sub-Saharan Africa (SSA) beginning in 2016, with South Africa among the earliest countries in the region to launch a national oral PrEP programme (65). Since then, countries including Kenya, Zimbabwe, Uganda, Tanzania, and Nigeria have progressively expanded access (55) (1). SSA continues to bear a disproportionate burden of the global HIV epidemic (57, 64), and PrEP has emerged as a cornerstone of the comprehensive combination prevention framework endorsed by the WHO, encompassing behavioral, biomedical, and structural strategies (56, 63) (1). Despite these foundational advances, the translation of PrEP policy into equitable, population-level uptake has remained inconsistent across SSA, revealing structural and systemic barriers that continue to undermine programme effectiveness.

In SSA, oral PrEP, primarily tenofovir disoproxil fumarate/emtricitabine (TDF/FTC), is delivered predominantly through primary healthcare (PHC) settings, including sexual and reproductive health (SRH) clinics, maternal and child health (MCH) services, family planning units, and HIV testing and counselling sites (1–3). Nurse-led, task-shifted delivery models have been central to scale-up, particularly in South Africa, Kenya, and Uganda (4–6). Despite WHO endorsement and national rollouts across more than 20 SSA countries, UNAIDS (2023) estimates that PrEP coverage remains substantially below the 10 million people targeted by 2025, with significant disparities across countries and between urban and rural settings. Adherence difficulties, pill fatigue, stigma, and frequent clinic visits have further constrained uptake and persistence, particularly among adolescent girls and young women, key populations, and other groups at substantial HIV risk (7, 8, 52).

These persistent implementation gaps have catalysed renewed interest in alternative PrEP modalities that may circumvent the adherence and stigma-related limitations inherent to daily oral regimens. Injectable PrEP, particularly long-acting cabotegravir (CAB-LA), offers a promising alternative (9, 10). CAB-LA, administered by intramuscular injection every 2 months, has demonstrated superior or equivalent protection compared to daily oral PrEP in major clinical trials (9, 11). The HPTN 083 trial among men who have sex with men and transgender women, and the HPTN 084 trial among cisgender women, both showed significant reductions in HIV incidence with CAB-LA compared to oral tenofovir disoproxil fumarate/emtricitabine. These findings position injectable PrEP as a potentially transformative innovation for HIV prevention in SSA (10, 12). Other PrEP modalities under investigation or early implementation include event-driven oral dosing (2–1-1 regimen), the dapivirine vaginal ring, and lenacapavir, a long-acting injectable requiring administration only twice yearly (every 6 months) (51, 58, 59).

Despite this promise, the introduction and scale-up of injectable PrEP in SSA face substantial implementation challenges (14). These include the need for cold-chain infrastructure to maintain medication integrity, injection-competent healthcare providers, appropriate private clinical spaces for administration, updated national clinical guidelines and standard operating procedures, robust supply chain management systems, and sustainable financing mechanisms (14, 15). Moreover, as with any health innovation, successful implementation depends critically on the readiness, knowledge, attitudes, and working conditions of frontline healthcare workers who will deliver these services (16). Against this backdrop, the present review seeks to critically examine the available evidence on long-acting injectable PrEP, particularly cabotegravir (CAB-LA), within the SSA context, assessing both its transformative potential and the implementation realities that will determine its public health impact.

Healthcare workers serve as the critical interface between biomedical innovations and the communities they serve (17). Their perceptions, technical competencies, confidence levels, organizational support, and working conditions directly influence both the quality-of-service provision and client acceptance. Understanding HCW perspectives is therefore essential for designing implementation strategies that are contextually appropriate, feasible, and sustainable (18). Healthcare workers’ knowledge about PrEP derives from diverse sources, including pre-service medical and nursing education, in-service training through national programs and implementing partners, participation in demonstration projects, informal peer learning, and clinical guidelines when available (19, 20). This heterogeneity in knowledge acquisition contributes to variable awareness and competence across cadres, settings, and countries (19–21).

Two recent systematic reviews directly address HCW perspectives in Africa/LMICs. Zhang et al. (22) synthesized 14 qualitative studies on HCW experiences implementing PrEP in low- and middle-income countries, extracted 122 findings, and identified barriers, facilitators, and recommendations. Femi-Lawal et al. (19) conducted a meta-analysis of 34 studies across 12 African countries, finding high PrEP awareness (85%) but poor knowledge (18%) among HCWs, with moderate positive attitudes (46%) and 58% willingness to prescribe.

However, both reviews noted significant limitations. Zhang et al. (22) rated the overall evidence quality as “low” using the ConQual approach. Femi-Lawal et al. (19) emphasized that knowledge gaps and attitudinal barriers hinder implementation despite awareness. Thus, while HCW perspectives have been synthesized, the evidence base remains limited in quality and scope, supporting the need for continued research on context-specific HCW barriers and facilitators.

Few published studies have examined HCW perspectives on injectable PrEP specifically, as most countries are only beginning to introduce it through demonstration projects. Mbatha et al. (23) explicitly state that “perspectives of primary healthcare workers…remain underexplored,” motivating their scoping review protocol. Only a handful of studies specifically address injectable PrEP and HCW perspectives. However, implementation experiences with oral PrEP provide critical insights into health system readiness, HCW training needs, service delivery models, and barriers and facilitators that will directly inform the rollout of injectable PrEP (14). Many implementation considerations, including task-shifting approaches, integration strategies with sexual and reproductive health or maternal and child health services, client counseling frameworks, stigma reduction efforts, and health system strengthening needs, transfer across PrEP modalities, as both formulations operate within the same health system contexts and face similar structural challenges (14, 16, 24).

Therefore, this narrative review examined HCW perspectives on PrEP delivery broadly, synthesizing evidence from both oral and injectable PrEP experiences, with focused synthesis on implications for injectable formulation introduction. This approach enables us to: (1) understand current HCW awareness, knowledge, and attitudes toward PrEP as a biomedical prevention technology; (2) identify common implementation barriers and facilitators relevant across formulations; (3) map training and capacity-building needs; (4) assess health system readiness factors; and (5) derive lessons from oral PrEP implementation that can inform more effective injectable PrEP rollout strategies. Taken together, the foregoing historical trajectory, current implementation challenges, and the emergence of long-acting injectable alternatives establish the rationale and urgency for the present review, whose methods are described in the following section.

## 2 Methods

### 2.1 Study design and approach

This study adopted a narrative review design. A thorough and transparent approach was applied to literature identification, selection, and synthesis to ensure credibility and coherence. This approach is particularly suitable for examining healthcare worker perspectives across diverse contexts and study designs in SSA, where evidence is still evolving and where both qualitative and quantitative findings contribute valuable implementation insights.

### 2.2 Eligibility criteria

#### 2.2.1 Inclusion criteria

*Population:* Studies involving healthcare workers, including doctors, nurses, pharmacists, clinical officers, counselors, and community health workers working in HIV prevention, sexual and reproductive health, maternal and child health, or related services in Sub-Saharan Africa.

*Intervention/focus:* Studies addressing healthcare workers’ knowledge, perceptions, attitudes, readiness, or experiences delivering or implementing HIV PrEP services. Studies examining oral PrEP (tenofovir-based regimens), injectable PrEP (cabotegravir), or PrEP broadly, without specifying formulation, were eligible.

*Geographic focus:* Studies conducted in Sub-Saharan African countries.

*Study design:* Primary research using qualitative (in-depth interviews, focus group discussions, ethnographic studies), quantitative (surveys, cross-sectional studies), or mixed methods approaches.

*Language:* Published in English.

*Time period:* Published between 2020 and 2025, reflecting the period during which PrEP implementation expanded across SSA and evidence on provider perspectives began accumulating.

#### 2.2.2 Exclusion criteria

Studies that focused exclusively on client or community perspectives without including healthcare worker data were excluded. Non-empirical publications such as systematic reviews, scoping reviews, commentaries, editorials, protocols, and policy documents without original primary data were excluded. Studies conducted outside SSA or published in languages other than English were not considered. Grey literature, including conference abstracts, unpublished dissertations, and program reports, was excluded. Studies were not excluded based on quality assessment scores, as methodological diversity in this emerging field provides valuable contextual insights and reflects the pragmatic realities of implementation research. However, methodological strengths and limitations of all included studies were carefully noted during data extraction and considered during synthesis.

#### 2.2.3 Rationale for including oral PrEP studies to inform injectable PrEP implementation

While our primary interest is injectable PrEP implementation, few published studies have specifically examined healthcare workers’ perspectives on injectable formulations in SSA, as most countries are only beginning to introduce them through demonstration projects. Therefore, we included studies examining HCW perspectives on PrEP delivery broadly, regardless of formulation, with a focused synthesis of lessons applicable to the injectable PrEP rollout. This approach was justified for several reasons:

*Limited injectable PrEP evidence:* Injectable PrEP is an emerging technology in SSA with limited implementation experience and published research on provider perspectives to date.

*Transferable implementation lessons:* Healthcare worker perspectives on oral PrEP implementation reveal broader patterns regarding readiness to deliver biomedical prevention innovations, professional norms regarding HIV prevention services, and systemic barriers (training gaps, infrastructure limitations, workload constraints, policy gaps) that apply across PrEP modalities.

*Common implementation considerations:* Many implementation factors transfer across PrEP formulations, including task-shifting approaches, service integration strategies with sexual and reproductive health or maternal and child health platforms, client counselling frameworks, stigma reduction efforts, and health system readiness requirements.

*Shared health system context:* Both oral and injectable PrEP operate within the same health system contexts in SSA and face similar structural challenges, including provider capacity, supply chain management, policy development, and resource allocation. Understanding how these factors have affected oral PrEP implementation directly informs planning for the introduction of injectable PrEP.

During synthesis, findings were analyzed with explicit attention to their implications for injectable PrEP implementation, including considerations unique to long-acting formulations, such as injection administration competencies, cold-chain requirements, logistics for bimonthly scheduling, and management of prolonged pharmacologic effects.

### 2.3 Literature search strategy

A comprehensive and systematic literature search was conducted across five electronic databases: PubMed (MEDLINE), Scopus, Web of Science, EBSCOhost (including Academic Search Complete and CINAHL), and Google Scholar. The search was conducted between May and June 2025 to capture the most recent publications. The lead reviewer (NNM) developed the search strategy with methodological guidance from a research librarian. The search strategy combined four concept blocks using Boolean operators. The first block included PrEP-related terms: “pre-exposure prophylaxis” OR “PrEP” OR “cabotegravir” OR “CAB-LA” OR “long-acting PrEP” OR “injectable PrEP” OR “tenofovir” OR “TDF” OR “emtricitabine” OR “FTC” OR “Truvada” OR “Descovy.” The second block captured healthcare worker terms: “healthcare worker*” OR “health worker*” OR “healthcare provider*” OR “health provider*” OR “nurse*” OR “doctor*” OR “physician*” OR “clinician*” OR “pharmacist*” OR “counselor*” OR “counsellor*” OR “community health worker*.” The third block included outcome-related terms: “perception*” OR “attitude*” OR “knowledge” OR “awareness” OR “readiness” OR “willingness” OR “view*” OR “perspective*” OR “experience*” OR “barrier*” OR “facilitator*” OR “implementation” OR “acceptability.” The fourth block specified geographic scope: “Sub-Saharan Africa” OR “Africa” OR individual country names (South Africa, Kenya, Uganda, Zimbabwe, Tanzania, Nigeria, etc.). All concept blocks were combined using the AND operator. Medical Subject Headings (MeSH) were utilized in PubMed to enhance search sensitivity and specificity. The search was limited to English-language publications from January 2020 to June 2025. No restrictions were placed on study design during the search phase. Additional studies were identified through manual screening of reference lists from included articles and relevant systematic reviews.

All retrieved citations were exported to EndNote 20 (Clarivate Analytics, US) for citation management. Duplicate records were identified and removed using EndNote’s automated deduplication function, supplemented by manual verification to ensure accuracy. The final deduplicated citation library was used for subsequent screening stages.

### 2.4 Study identification and selection

Literature identification and selection proceeded through three stages: title review, abstract review, and full-text assessment. The lead reviewer (NNM) conducted all screening stages with methodological guidance from a research librarian who assisted with database search strategies and retrieval processes. Predefined scope criteria guided decisions at each stage.

In the first phase, titles of all retrieved records were screened to identify and exclude obviously irrelevant studies (e.g., studies not conducted in SSA, not focusing on HCWs, or addressing topics unrelated to PrEP). In the second phase, abstracts of remaining articles were screened against the full eligibility criteria to assess potential relevance. Studies that clearly did not meet the inclusion criteria were excluded at this stage, while those with insufficient information in the abstract were retained for full-text assessment. In the final phase, full-text articles were retrieved and reviewed comprehensively to determine final eligibility. Studies excluded at the full-text stage were documented with specific reasons.

**Figure 1:**
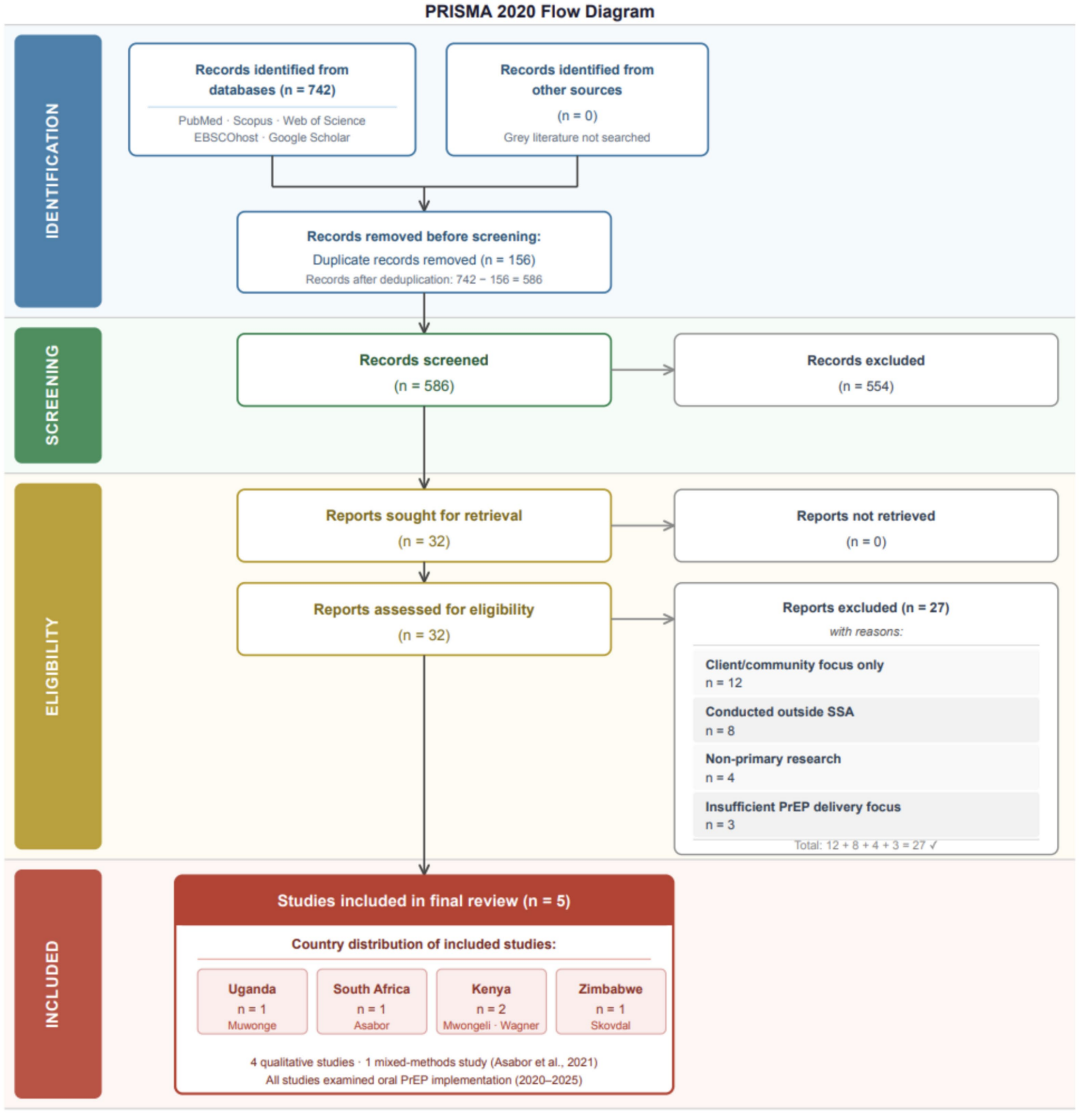
PRISMA 2020 flow diagram depicting the systematic literature search and study selection process. Records were identified across five databases (PubMed, Scopus, Web of Science, EBSCOhost, and Google Scholar). Following the removal of 156 duplicates, 586 records underwent title and abstract screening. Thirty-two full-text articles were assessed for eligibility; exclusions by reason: client/community focus only (*n =* 12); conducted outside SSA (*n =* 8); non-primary research (*n =* 4); insufficient PrEP delivery focus (*n =* 3). Five studies met all scope criteria and were included in the final synthesis.

Throughout the selection process, any uncertainties regarding study eligibility were discussed with the supervisory team (NM and DG) to ensure consistent application of the scope criteria. The selection process is summarised in Figure 1, which shows the number of records identified, reviewed, and included at each stage, along with reasons for exclusion.

### 2.5 Data review and synthesis preparation

Relevant information from included studies was reviewed and organised to facilitate thematic synthesis. The lead reviewer (NNM) conducted an in-depth reading of each included study, extracting relevant information into a structured framework aligned with the review objectives. Extracted information and emerging interpretations were discussed with the supervisory team (NM and DG) throughout the process to ensure completeness and accuracy.

For each study, key information was noted, including study characteristics (author, year, country, setting, design, methods, sample composition) and substantive findings related to healthcare workers’ perspectives on PrEP delivery. The review focused on identifying findings across five thematic areas: awareness and knowledge of PrEP, attitudes and perceptions toward PrEP as a prevention strategy, readiness and willingness to deliver PrEP services, barriers to implementation at individual, facility, and health system levels, and facilitators or enabling conditions supporting successful delivery. Information on the PrEP formulation in each study (oral, injectable, or unspecified) was also noted to inform synthesis of the implications for injectable PrEP implementation.

The review proceeded iteratively, with continuous comparison across studies to identify recurring patterns, contrasts, and unique contextual factors. This approach preserved the nuance and context essential for meaningful synthesis of evidence from diverse settings and methodological approaches and formed the foundation for the narrative thematic synthesis described in Section 2.7.

### 2.6 Quality assessment

The lead reviewer conducted a quality assessment to evaluate the methodological rigor and report quality of the included studies, using criteria adapted from the Consolidated Criteria for Reporting Qualitative Research (COREQ; (25)). Each study was assessed against key criteria adapted for qualitative research, including: (1) clarity of research aims and objectives; (2) appropriateness of study design and methods for addressing research questions; (3) relevance to the review questions; and (4) transparency and clarity of reporting of methods and findings.

Additional qualitative quality dimensions were noted during assessment, including sampling strategies, use of theoretical or conceptual frameworks, data collection approaches (interview guides, focus group protocols), analytical rigor (coding procedures, thematic analysis methods), and quality enhancement strategies such as reflexivity statements, triangulation, and member checking or validation procedures.

Studies were not excluded based on quality scores, as methodological diversity provides valuable contextual insights in this emerging field. However, the methodological strengths and limitations of the included studies were documented and considered during synthesis to assess confidence in findings.

### 2.7 Thematic synthesis

Findings from included studies were synthesized using a narrative thematic approach, which allows for in-depth exploration of healthcare workers’ perspectives across diverse contexts and study designs. Studies were read closely to identify recurring concepts and patterns, which were grouped into themes through an iterative process of comparison and categorization. During synthesis, particular attention was paid to identifying findings with direct implications for injectable PrEP rollout. The synthesis specifically examined evidence related to healthcare worker training and capacity-building requirements, infrastructure and supply chain considerations, task-shifting and staffing models, client counseling approaches, service integration strategies, and broader health system readiness factors. This focused approach to synthesis was designed to ensure that the review would provide actionable insights for injectable PrEP implementation planning, moving beyond general PrEP evidence to address the specific requirements and considerations relevant to long-acting injectable formulations. The narrative synthesis approach was selected as most appropriate given the heterogeneity of included studies in terms of design, geographic context, and healthcare worker populations. This approach enabled a comprehensive understanding of factors influencing PrEP delivery and uptake within the SSA healthcare context while maintaining attention to contextual variations and implementation nuances that quantitative meta-analysis would not capture.

## 3 Results

### 3.1 Study selection and characteristics

A literature search across the five databases identified 742 records. After removing 156 duplicates, 586 records were reviewed at the title and abstract level. Of these, 32 full-text articles were assessed for relevance. Five primary research studies met the scope criteria and were included in the final synthesis.

The most common reasons for full-text exclusion were focus on client or community perspectives rather than HCW perspectives (*n =* 12), conducted outside SSA (*n =* 8), non-primary research including systematic reviews and protocols (*n =* 4), and insufficient focus on PrEP delivery perspectives or primarily focused on treatment rather than prevention (*n =* 3).

### 3.2 Characteristics of included studies

The five included studies were conducted in four countries across SSA: Uganda (20) (*n =* 1), South Africa (26) (*n =* 1), Kenya (16, 27) (*n =* 2), and Zimbabwe (28) (*n =* 1). Four of the five studies employed qualitative designs using in-depth interviews or focus group discussions with healthcare workers; Asabor et al. (26) employed a convergent mixed-methods design comprising a structured quantitative survey administered to *n =* 215 healthcare workers and in-depth qualitative interviews with *n =* 24 healthcare workers, enabling integration of statistical breadth and interpretive depth. Sample sizes across qualitative studies ranged from *n =* 18 to *n =* 24 participants drawn from frontline provider populations in specific clinic settings.

Notably, all five included studies examined oral PrEP implementation experiences. No studies specifically examining injectable PrEP implementation met the review’s scope criteria, reflecting that injectable formulations are only recently being introduced in SSA, and published research on HCW perspectives remains limited. However, as discussed in our methods, oral PrEP implementation experiences provide critical insights directly applicable to injectable PrEP rollout (Table 1).

**Table 1:** Characteristics of included studies.

Author, year/country	Setting and design	Study objectives	Sample	Key findings
Muwonge et al. (20) Uganda	Urban public health facilities; Qualitative (IDIs, FGDs)	To explore HCW perspectives on PrEP service delivery in central Uganda, including awareness, attitudes, barriers, and facilitators.	HCWs (*n =* 24)	Higher awareness in PrEP-programme facilities. Positive attitudes, persistent protocol, and side-effect management knowledge gaps. Mentorship is identified as a key enabler.
Asabor et al. (26) South Africa	Rural primary care clinics; Convergent mixed-methods	To assess HCW knowledge, attitudes, and practices regarding PrEP delivery in rural South African PHC settings using a mixed-methods design.	Quantitative survey: *n =* 215 HCWs; Qualitative IDIs: *n =* 24 HCWs	Quantitative: 58% adequate PrEP knowledge; 72% willing to prescribe; nurses scored significantly higher than doctors (p < 0.05). Qualitative: Knowledge gaps in rural facilities; PrEP/PEP/ART confusion; supportive attitudes; need for structured training.
Mwongeli et al. (27) Kenya	MCH clinics; Qualitative (IDIs)	To examine MCH provider perspectives on integrating PrEP into antenatal and postpartum care in Kenya.	MCH providers (*n =* 18)	Strong support for PrEP-MCH integration; pregnancy framed as a heightened vulnerability window. Barriers: heavy workloads, competing priorities, and limited counselling training.
Wagner et al. (16) Kenya	SRH/family planning services; Qualitative (IDIs, FGDs)	To identify implementation determinants and strategies for PrEP integration into SRH/family planning services from frontline provider perspectives.	Frontline providers (*n =* 22)	Task-shifting willingness among nurses and counsellors. PrEP-FP service synergies identified. Barriers: absent national guidelines, infrastructure constraints, and workload. Peer learning is valued.
Skovdal et al. (28) Zimbabwe	Primary healthcare centres; Qualitative (IDIs)	To document HCW recommendations for improving PrEP access for adolescent girls and young women in eastern Zimbabwe.	Healthcare providers (*n =* 20)	Willingness to provide PrEP to AGYW. Concerns: limited adolescent SRH counselling experience, confidentiality and consent for minors, community awareness needs.

HCW, healthcare worker; IDI, in-depth interview; FGD, focus group discussion; MCH, maternal and child health; SRH, sexual and reproductive health; FP, family planning; PHC, primary healthcare; AGYW, adolescent girls and young women; PrEP, pre-exposure prophylaxis.

### 3.3 Quality assessment

All five included studies met the quality assessment criteria, demonstrating clear research aims, appropriate methodological designs, relevance to the review questions, and transparent reporting of methods and findings. Studies employed rigorous qualitative methods including purposive sampling strategies, semi-structured interview guides informed by implementation science frameworks, iterative data collection and analysis, and systematic coding and thematic analysis procedures. Most studies reported the use of established qualitative quality frameworks and included reflexivity statements regarding researcher positionality.

Methodological strengths included the use of theoretical frameworks (e.g., the Consolidated Framework for Implementation Research), triangulation through multiple data collection methods (interviews and focus groups), and member checking or validation strategies with participants. Limitations noted across studies included potential selection bias toward more engaged or motivated HCWs, limited generalizability beyond specific clinic settings or regions, and, in some cases, small sample sizes that may not capture the full diversity of perspectives. The full quality assessment instrument and completed per-study appraisals are publicly available at: FIGSHARE (23).

### 3.4 Main findings

The studies included in this review identified five main thematic areas influencing PrEP implementation in SSA from healthcare workers’ perspectives: (1) awareness and knowledge of PrEP; (2) attitudes and perceptions toward PrEP; (3) readiness and willingness to implement PrEP services; (4) barriers to service delivery; and (5) facilitators and enabling conditions. Each theme is presented below.

#### 3.4.1 Awareness and knowledge of PrEP

Healthcare workers across the included studies demonstrated varied levels of awareness and knowledge regarding PrEP, with differences based on prior exposure to PrEP programming, geographic location, and facility type.

Muwonge et al. (20) in Uganda found that HCWs working in facilities implementing PrEP demonstration projects in Uganda had substantially higher awareness and knowledge compared to providers in facilities without such programs. Providers familiar with PrEP could articulate basic eligibility criteria, dosing schedules, and monitoring requirements for oral PrEP. However, knowledge gaps persisted regarding specific clinical protocols, management of side effects, and risk assessment procedures.

Asabor et al. (26) documented significant knowledge gaps among rural primary care providers in South Africa. In the quantitative component (*n =* 215), only 58% of surveyed HCWs reported adequate PrEP knowledge, and nurses scored significantly higher on PrEP knowledge assessments than doctors (*p* < 0.05). The qualitative component (*n =* 24 IDIs) corroborated these findings: many HCWs in facilities without pilot programme exposure had heard of PrEP only through informal channels such as media or peer discussions but lacked structured training on clinical protocols, contraindications, or counselling approaches. Some providers confused PrEP with post-exposure prophylaxis (PEP) or antiretroviral treatment (ART).

Wagner et al. (16) in Kenya found that HCWs working in sexual and reproductive health and family planning services in Kenya had variable knowledge, with nurses and counselors often having better practical knowledge than doctors. Operational knowledge regarding integrating PrEP into existing workflows, documentation requirements, and supply management remained inconsistent.

Skovdal et al. (28) reported that knowledge about delivering PrEP to specific populations, particularly adolescent girls and young women, was limited in Zimbabwe. Providers expressed uncertainty about how to counsel young clients, obtain appropriate consent, maintain confidentiality, and address concerns about sexual behavior disinhibition.

#### 3.4.2 Attitudes and perceptions toward PrEP

HCWs across studies generally expressed positive attitudes toward PrEP as a biomedical prevention tool, recognizing its potential to expand HIV prevention options for at-risk populations.

Muwonge et al. (20) documented that Ugandan HCWs viewed PrEP favorably, particularly for key populations including female sex workers, serodiscordant couples, and young women in high-prevalence settings. Providers noted that PrEP offered an additional layer of protection beyond condoms and could be user-initiated.

Asabor et al. (26) provide important cadre-level insights into healthcare workers’ attitudes towards PrEP. The quantitative component (*n =* 215) included both prescribers (doctors and professional nurses) and non-prescribers (e.g., staff nurses, counsellors, and other cadres), with prescribers comprising approximately 30% of the sample. The qualitative component (*n =* 24) consisted exclusively of nurses, including both professional and staff nurses, all of whom had substantial experience in HIV care. Qualitative findings indicated that nurses expressed a strong sense of professional ownership over PrEP delivery, positioning it as an extension of their existing HIV and sexual health responsibilities. These cadre distinctions are important for informing task-shifting strategies and workforce training as PrEP implementation expands.

Mwongeli et al. (27) reported that maternal and child health providers in Kenya strongly supported integrating PrEP into antenatal and postpartum care. These providers emphasized that pregnancy and the postpartum period represent windows of heightened HIV vulnerability, and offering PrEP could protect both mothers and infants.

Wagner et al. (16) documented positive attitudes toward PrEP integration with family planning services, with providers noting natural synergies in target populations, counseling approaches, and service delivery platforms. Some providers viewed PrEP as a means of addressing the dual concerns of unintended pregnancy and HIV acquisition among young women.

Skovdal et al. (28) found that providers in Zimbabwe expressed enthusiasm about offering PrEP to adolescent girls and young women but also voiced ethical concerns about providing prevention services to minors, navigating parental consent requirements, and ensuring clients fully understood the long-term commitment required for adherence.

#### 3.4.3 Readiness and willingness to deliver PrEP

Healthcare workers across studies expressed general willingness to deliver PrEP services, describing it as part of their professional responsibility in HIV prevention. However, readiness varied considerably based on training received, resource availability, and organizational support.

Muwonge et al. (20) found that HCWs in facilities with PrEP programs in Uganda felt reasonably prepared to deliver services after receiving training and ongoing mentorship. Providers in facilities without established programs expressed hesitation, citing a lack of training, unclear guidelines, and concerns about managing potential side effects or drug interactions without specialist support.

Asabor et al. (26) documented that, despite 72% of surveyed HCWs (*n =* 215) expressing willingness to prescribe PrEP, rural South African providers felt ill-equipped due to insufficient training and absence of clinical support systems. Importantly, nurses reported significantly higher readiness than doctors, attributing this to their closer engagement with patients’ daily health needs and their existing roles in HIV testing and reproductive health counselling. Many HCWs expressed concern about taking on additional responsibilities without corresponding staffing increases or resource allocation.

Wagner et al. (16) found strong willingness among nurses and counselors in Kenya to deliver PrEP through task-shifting arrangements, describing it as a logical extension of their existing roles in HIV testing, family planning, and sexual health counseling. They emphasized the need for clear scope-of-practice guidelines, supportive supervision, and mechanisms for referring complex cases to clinicians.

Mwongeli et al. (27) reported that maternal and child health providers in Kenya were willing to integrate PrEP into their services but identified significant barriers, including already heavy workloads, competing priorities in antenatal care, and the need for additional training in PrEP-specific counseling for pregnant and postpartum women.

Skovdal et al. (28) documented willingness to provide PrEP to adolescent girls and young women in Zimbabwe but noted that readiness was constrained by limited experience with adolescent sexual health counseling, concerns about confidentiality and consent processes, and the need for youth-friendly service delivery training.

#### 3.4.4 Barriers to PrEP implementation

Healthcare workers identified multiple interconnected barriers spanning individual, facility, and health system levels.

Training and knowledge gaps emerged as a primary barrier across all studies. Providers consistently emphasized the lack of formal training in PrEP protocols, eligibility assessment, side-effect management, and client counseling. Muwonge et al. (20) noted that even when training occurred, it was often a one-off workshop without ongoing refresher training or supportive supervision. Asabor et al. (26) found that knowledge gaps led to provider hesitation and inconsistent service delivery.

Inadequate infrastructure and supplies represented another critical barrier. Wagner et al. (16) and Skovdal et al. (28) documented challenges with medication stockouts, unreliable supply chains, and a lack of point-of-care HIV testing supplies necessary for PrEP initiation and monitoring. Several studies noted that facilities lacked private counseling spaces.

Workload and staffing constraints featured prominently. Mwongeli et al. (27) found that integrating PrEP into already-busy maternal and child health services was challenging due to time pressures and competing demands. Providers worried that adding PrEP without additional staff would compromise the quality of care across all services. Asabor et al. (26) documented concerns about burnout and inadequate staffing levels to accommodate expanded service offerings.

Lack of clinical guidelines and protocols created uncertainty and inconsistency. Multiple studies noted the absence of nationally endorsed, context-specific PrEP guidelines adapted to local realities. This left providers uncertain about standard procedures, documentation requirements, and how to handle clinical scenarios not explicitly addressed in training.

Stigma and confidentiality concerns emerged as barriers affecting both HCW comfort in delivering PrEP and client willingness to access services. Skovdal et al. (28) found that some providers worried about being associated with services for key populations or young sexually active individuals due to community stigma. Wagner et al. (16) noted that integrated service delivery could inadvertently signal PrEP use to other clients, raising confidentiality concerns.

Limited community awareness and demand were identified as a barrier. Providers noted that clients’ limited knowledge of PrEP reduced uptake, even when services were available. Muwonge et al. (20) emphasized the need for community education and demand creation to complement provider readiness.

#### 3.4.5 Facilitators and enabling conditions

Studies identified several facilitators that supported PrEP implementation.

Task-shifting and role clarification emerged as a key facilitator. Wagner et al. (16) found that clearly defining roles and empowering nurses and lay counselors to deliver PrEP services (with appropriate training and supervision) expanded access and reduced burden on doctors. Providers responded positively to task-shifting when accompanied by supportive supervision and referral mechanisms for complex cases.

Service integration facilitated PrEP uptake. Mwongeli et al. (27) demonstrated that integrating PrEP into maternal and child health services normalized it as part of comprehensive healthcare rather than a standalone HIV prevention service. Wagner et al. (16) found that integration with family planning services created natural entry points for PrEP counseling and initiation.

Structured training and mentorship were consistently identified as enabling successful implementation. Muwonge et al. (20) noted that facilities with ongoing mentorship and supportive supervision from experienced providers or program staff demonstrated better PrEP delivery quality and provider confidence compared to facilities relying solely on one-time training.

Government commitment and policy support facilitated implementation. Studies conducted in settings with national PrEP guidelines, government endorsement, and budget allocation for PrEP commodities reported more systematic implementation compared to settings where PrEP remained mainly donor-funded or project-based.

Peer learning and provider networks enabled knowledge sharing and problem-solving. Asabor et al. (26) and Skovdal et al. (28) documented that providers valued opportunities to learn from peers implementing PrEP in similar settings, exchange practical strategies, and troubleshoot challenges collectively.

Client demand and positive experiences motivated providers. Muwonge et al. (20) found that seeing clients benefit from PrEP, particularly clients who had been at high risk and successfully remained HIV-negative, reinforced provider commitment to service delivery.

Demonstration projects and implementation support provided learning opportunities. Multiple studies noted that facilities participating in demonstration projects or receiving implementation support (training, mentorship, commodity supplies, monitoring systems) achieved more successful integration than facilities attempting implementation without external support.

## 4 Discussion

This narrative review synthesized healthcare workers’ perspectives on PrEP delivery in SSA to inform the implementation of injectable PrEP. While all included studies examined oral PrEP, the implementation lessons directly transfer to injectable formulations, with many challenges likely intensified given additional technical requirements.

### 4.1 Knowledge gaps signal extensive training needs

HCWs demonstrated variable PrEP knowledge strongly mediated by prior exposure to structured programs. Providers in demonstration project facilities showed substantially better understanding than those learning through informal channels, with some confusing PrEP with PEP or ART. This pattern reflects established implementation science principles: passive awareness does not translate to clinical competency without structured training, hands-on practice, and ongoing mentorship (29, 30). Knowledge gaps extended beyond biomedical facts to operational understanding of workflow integration, risk assessment, and counseling approaches, particularly for adolescent populations (31).

The field of long-acting HIV prevention is evolving rapidly, with the PURPOSE 1 trial demonstrating 100% efficacy of twice-yearly subcutaneous lenacapavir among cisgender women in Uganda and South Africa (58) and PURPOSE 2 showing a 96% reduction in HIV incidence among men and gender-diverse persons (59). Lenacapavir received FDA approval in June 2025 (51), and several SSA countries have initiated rollout. These developments reinforce the central argument of this review: the window for systematic preparation of healthcare workers is narrow, and research must keep pace with programmatic rollout.

For injectable PrEP, these knowledge deficits signal substantial preparatory work required. The specialized competencies needed, injection technique, cold-chain management, and managing prolonged pharmacologic effects represent expanded training domains beyond oral PrEP. Evidence from injectable contraceptive programs demonstrates that successful introduction requires comprehensive, competency-based training addressing not just technical skills but counseling about long-acting interventions and managing discontinuation scenarios (32, 33).

### 4.2 Positive attitudes tempered by implementation concerns

Healthcare workers expressed generally positive attitudes toward PrEP, recognizing its potential to expand prevention options for high-risk populations. Strong support emerged for integration with maternal and child health and family planning services, reflecting provider recognition of heightened HIV vulnerability during pregnancy and postpartum periods (34, 35). However, concerns about behavioral disinhibition persisted despite evidence from systematic reviews finding no meaningful risk compensation among PrEP users (36, 37). These concerns often reflected normative judgments about sexual behavior rather than evidence-based assessment (36), requiring interventions addressing underlying values alongside information provision.

For injectable PrEP, ethical concerns intensify given the long-acting nature and inability to discontinue immediately. Ensuring informed consent requires counseling protocols addressing the extended pharmacologic tail, continued need for other prevention methods, and what committing to bimonthly injections entails, considerations parallel to those for long-acting reversible contraception (38).

### 4.3 Conditional readiness requires systems support

Healthcare workers expressed willingness to deliver PrEP, but readiness varied dramatically based on training, resources, and organizational support. This conditional nature reflects implementation science principles: individual motivation cannot compensate for organizational and systems-level barriers (39, 40). Workload concerns were particularly prominent, with providers worried that adding PrEP without corresponding staffing increases would compromise all services, a legitimate concern given that healthcare worker workload in SSA often exceeds sustainable levels (41).

Asabor et al. (26) provide particularly instructive cadre-level evidence on readiness differentials. Quantitative data (*n =* 215) demonstrated that nurses scored significantly higher on PrEP knowledge than doctors (*p* < 0.05) and expressed greater willingness to prescribe (72% overall, with nurses disproportionately driving this figure). Qualitative data (*n =* 24 IDIs) revealed the underlying logic: nurses and counsellors articulated PrEP delivery as a natural extension of their existing HIV testing and reproductive health roles, whereas doctors more frequently cited scope-of-practice uncertainty, time constraints, and concerns about managing adverse events without specialist backup. These findings have direct implications for task-shifting policy design: training investments and role clarification should prioritize the nurse and counsellor cadres who demonstrate the strongest readiness and the closest alignment with the service delivery contexts in which PrEP will be integrated.

Task-shifting to nurses and counselors emerged as both necessary and effective when accompanied by clear guidelines, training, and supervision. Extensive evidence supports task-shifting for HIV services, with nurses achieving outcomes comparable to doctors when appropriately prepared (42, 43). For injectable PrEP, nurse administration will be essential given physician shortages, with precedent from injectable contraceptive programs demonstrating safety and effectiveness (33).

However, task-shifting itself remains a contested strategy, and the manuscript’s endorsement of it warrants critical contextualisation. A persistent tension exists between task-shifting as a pragmatic necessity driven by physician shortages and task-shifting as a potential driver of professional deskilling, role ambiguity, and overburdening of lower-cadre workers who are already stretched (60, 61). Qualitative evidence from LMICs documents that nurses and community health workers frequently perceive task-shifting as cost-cutting rather than genuine scope expansion, leading to resentment, burnout, and high attrition among the very cadres on whom expanded PrEP delivery depends (66). Regulatory frameworks governing scope-of-practice remain highly fragmented across SSA, with task-shifting reforms described as “often lengthy and controversial” even in high-income settings (62). For injectable PrEP specifically, questions of who is authorised to administer intramuscular or subcutaneous injections, initiate PrEP, and manage adverse events will require country-specific regulatory decisions that cannot be assumed to follow oral PrEP precedent. Successful task-shifting models must therefore address not only training and supervision but also formal scope-of-practice legislation, commensurate remuneration, and workload protections, conditions that remain inconsistently met across the region.

### 4.4 Systemic barriers demand comprehensive solutions

Multiple interconnected barriers emerged, spanning training gaps, infrastructure inadequacies, supply chain vulnerabilities, staffing constraints, absent guidelines, stigma, and limited community awareness. These reflect broader health system fragilities in SSA rather than PrEP-specific problems (44, 53, 54). For injectable PrEP, cold-chain requirements will intensify infrastructure challenges, as cabotegravir requires refrigeration, creating additional constraints (14). The absence of national guidelines creates uncertainty and inconsistency, requiring policy development well before implementation (45).

Provider stigma and concerns about stigma by association represent persistent challenges requiring multi-level interventions including education, supportive organizational cultures, and broader social change efforts (46). Integration strategies positioning injectable PrEP within comprehensive sexual and reproductive health services may reduce stigma while creating confidentiality challenges requiring careful service design (16, 47).

### 4.5 Facilitators provide implementation roadmap

Key facilitators consistently supporting implementation included task-shifting with clear role definitions, service integration with existing platforms, structured training with ongoing mentorship, government commitment and policy support, peer learning networks, and demonstration projects building evidence and capacity. These findings align with implementation science frameworks emphasizing multi-level strategies (48).

Government commitment will be especially critical given cabotegravir’s higher costs compared to oral PrEP, requiring domestic financing and policy support for sustainable scale-up (49). Demonstration projects should generate actionable implementation knowledge through systematic documentation before broader rollout (50).

For injectable PrEP specifically, primary healthcare settings represent the most appropriate and equitable delivery platform. The evidence from oral PrEP implementation consistently demonstrates that integration into existing PHC-based SRH, MCH, and family planning services, rather than vertical, facility-specific HIV programmes, normalizes PrEP as part of routine healthcare and reaches women, adolescents, and key populations at existing service touchpoints. Injectable PrEP delivery should follow the same logic, with bimonthly injection visits aligned with routine PHC appointment cycles (e.g., quarterly depot contraceptive visits). In terms of provider cadre, NIMART-trained nurses (Nurse-Initiated Management of Antiretroviral Therapy) and PIMART-trained pharmacists (Pharmacist-Initiated Management of Antiretroviral Therapy) represent the most feasible task-shifted delivery cadre in SSA countries where these frameworks exist, given their established scope of practice for ART initiation and their demonstrated competency with injectable administration in contraceptive programmes. HIV specialist clinicians, including HAST nurses and HIV specialist doctors, should serve as referral and supervisory resources for complex cases and adverse event management rather than primary delivery agents, to preserve equitable geographic and systemic access.

### 4.6 Strengths, limitations, and implications

#### 4.6.1 Strengths

This narrative review offers several methodological and contextual strengths. The literature search was systematic and comprehensive, spanning five major electronic databases (PubMed, Scopus, Web of Science, EBSCOhost, and Google Scholar) with clearly defined eligibility criteria, yielding an initial pool of 742 records. The included studies span four SSA countries, Uganda, South Africa, Kenya, and Zimbabwe, providing geographic diversity that captures variation in health system contexts, PrEP programme maturity, and HCW cadre composition. The synthesis was conducted with explicit attention to the implications for injectable PrEP implementation, a distinction that differentiates this review from the two existing broader syntheses (19, 22). Quality assessment was conducted using adapted COREQ criteria, and included studies demonstrated overall methodological rigour, including the use of implementation science frameworks, purposive sampling, and established qualitative analytical procedures.

#### 4.6.2 Limitations

A notable limitation of this review is the small number of primary studies (*n =* 5) meeting the predefined scope criteria. We wish to be transparent that this reflects the current state of the peer-reviewed evidence base in SSA on this specific topic, not a deficiency of the search strategy: a comprehensive search across five major databases from 742 initial records, filtered by stringent eligibility criteria, yielded only five primary empirical studies that specifically examined HCW perspectives on PrEP delivery in SSA within the 2020–2025 period. The existing broader evidence base, synthesised in the systematic review by Zhang et al. (22) and the meta-analysis by Femi-Lawal et al. (19), is explicitly cited throughout this manuscript as the complementary broader foundation. Our contribution is a more focused narrative synthesis of SSA-specific primary studies, with explicit attention to injectable PrEP implications, a contribution distinct from and not duplicative of these existing reviews. Beyond the limited volume of primary studies, it is important to acknowledge the possibility of selection bias arising from the geographic and contextual distribution of the included studies. The five studies identified may disproportionately represent specific SSA countries or healthcare settings, particularly those with more established PrEP infrastructure, such as South Africa and Kenya, which could limit the generalisability of findings to lower-resourced or more epidemiologically diverse contexts within the region. Countries where injectable PrEP trials or implementation pilots are nascent or unpublished remain underrepresented in the available evidence base, and this structural gap in the literature is itself a significant finding. Consequently, conclusions regarding healthcare system readiness, community acceptability, and delivery feasibility should be interpreted with appropriate caution and may not be uniformly applicable across the heterogeneous health systems of SSA.

Beyond sample size, additional limitations include: the absence of any primary studies specifically examining HCW perspectives on injectable PrEP in SSA (requiring extrapolation from oral PrEP experiences that may not fully capture injectable-specific concerns); cross-sectional designs in all included studies, limiting causal inference and longitudinal understanding of readiness trajectories; limited attention to client experiences and community-level factors that interact with provider readiness; and the exclusion of grey literature, including conference abstracts, implementation reports, and programme evaluations, which may contain valuable implementation data not yet published in peer-reviewed journals. While prior systematic reviews have made important contributions to the PrEP literature in SSA, notably Zhang et al. (22), who examined HCW experiences implementing PrEP across low- and middle-income countries, and Femi-Lawal et al. (19), whose meta-analysis addressed HCW knowledge, attitudes, and willingness to prescribe PrEP across 12 African countries, neither review centred the implementation implications of long-acting injectable cabotegravir (CAB-LA) as their primary analytical focus. The present review is specifically oriented toward the PHC-level delivery considerations, nurse-led implementation feasibility, and health system readiness requirements that are prerequisite to CAB-LA scale-up in SSA. This distinct focus addresses an emerging and time-sensitive evidence gap: as CAB-LA transitions from clinical trial contexts into real-world programmatic deployment, evidence synthesising its implementation landscape at the primary care level is critically needed to inform rollout strategy, policy adaptation, and resource allocation. In this respect, the current synthesis offers a complementary rather than duplicative contribution to the existing literature.

#### 4.6.3 Research implications

The identification of only five eligible primary studies constitutes, in itself, a substantive finding: it reveals that peer-reviewed implementation research on HCW perspectives in SSA has not kept pace with the rapid programmatic rollout of both oral and injectable PrEP. This evidence gap is particularly acute for injectable formulations, for which no studies meeting the review’s scope criteria were identified. To facilitate strategic planning and guide future scholarly inquiry, the following research priorities are organised into short-term and longer-term agendas. In the short term, the most pressing research needs include: (1) implementation science studies examining the feasibility and acceptability of CAB-LA delivery within existing nurse-led PHC platforms, particularly in high-burden, resource-constrained settings across SSA; (2) mixed-methods investigations into community-level acceptability and demand generation, with specific attention to key populations and gender-disaggregated outcomes; and (3) health system readiness assessments evaluating cold-chain logistics, injection supply infrastructure, and HCW training requirements at the facility level. These priorities directly address the implementation gaps identified in this review and are most proximal to the current programmatic rollout timeline for CAB-LA and lenacapavir. Over the longer term, research should prioritise: (1) longitudinal cohort studies examining real-world adherence, retention, and virological outcomes under programmatic conditions, as distinct from controlled trial environments; (2) cost-effectiveness analyses comparing CAB-LA with oral TDF/FTC across diverse SSA health system typologies; (3) implementation trials co-designing and evaluating community health worker-mediated delivery models as a strategy to extend injectable PrEP reach into rural and peri-urban settings; and (4) cadre-specific task-shifting and scope-of-practice adaptation studies. Future systematic reviews should incorporate broader search strategies, including multiple languages, grey literature, and team-based screening and extraction protocols to minimise bias and maximize evidence capture. Establishing this tiered research agenda will enable evidence generation that is both methodologically responsive to current gaps and practically aligned with the programmatic timelines of national HIV prevention strategies across SSA.

#### 4.6.4 Policy and practice implications

For policy and practice, findings from this review consistently point to PHC-based delivery as the most equitable and scalable platform for injectable PrEP in SSA. Both the included studies and broader oral PrEP implementation evidence demonstrate that integration into existing SRH, MCH, and family planning services, rather than vertical HIV-specific programmes, normalises PrEP as part of routine healthcare and reaches priority populations at existing care touchpoints. Injectable PrEP delivery should follow this model, with bimonthly injection visits incorporated into routine PHC appointment cycles.

In terms of provider cadre, NIMART-trained nurses and PIMART-trained pharmacists represent the most feasible task-shifted delivery cadre, given their established scope of practice for ART initiation and their existing competency with injectable administration in contraceptive programmes (33, 43). General practitioners and specialist HIV clinicians should serve as referral and supervisory resources for complex cases, adverse event management, and counselling on the extended pharmacologic tail of long-acting formulations, not as primary delivery agents.

Policymakers across SSA should urgently review and, where necessary, update scope-of-practice legislation to explicitly authorise NIMART nurses and PIMART pharmacists to initiate and administer injectable PrEP. Preparation for the introduction of injectable PrEP must also include: national clinical guideline development and adaptation; competency-based pre-service and in-service training curricula; cold-chain infrastructure investment; robust supply chain management systems; sustainable domestic financing mechanisms; and community-level demand creation strategies. Demonstration projects with integrated implementation research components should precede broad rollout to generate context-specific evidence and systematically build health system capacity.

#### 4.6.5 Synthesis

Healthcare workers in SSA demonstrate positive attitudes but significant readiness gaps that will affect injectable PrEP implementation (16, 20, 26–28). Successful introduction requires multi-level strategies addressing training, task-shifting, service integration, infrastructure, policy, and financing, leveraging facilitators and mitigating barriers identified through oral PrEP implementation experiences.

## 5 Conclusion

This narrative review synthesised evidence on HCWs’ perspectives on HIV PrEP delivery in SSA to inform the introduction and scale-up of injectable PrEP formulations. Across five primary studies conducted in Uganda, South Africa, Kenya, and Zimbabwe, consistent patterns emerged: HCWs demonstrated positive attitudes toward PrEP as a biomedical prevention strategy but exhibited variable readiness shaped by training exposure, resource availability, cadre-specific roles, and the degree of organizational and policy support available. Nurses and lay counsellors consistently demonstrated stronger knowledge, more favourable attitudes, and greater willingness to deliver PrEP than doctors, underscoring the critical importance of task-shifting as a delivery strategy, provided that scope-of-practice regulations, training frameworks, and workload protections are concurrently addressed.

The identification of only five eligible primary studies reflects a substantive evidence gap: peer-reviewed implementation research on HCW perspectives in SSA has not kept pace with the rapid programmatic rollout of both oral and injectable PrEP. This gap is especially acute for injectable formulations, for which no primary studies met the review’s scope criteria. As CAB-LA are introduced across SSA, including in Zimbabwe, Zambia, and South Africa, there is an urgent need for country-specific implementation research on provider readiness, competency-based training models, and delivery system integration.

For policy and practice, this review identifies primary healthcare as the most appropriate and equitable delivery platform for injectable PrEP, with NIMART-trained nurses and PIMART-trained pharmacists serving as the most feasible provider cadre for task-shifted administration. Policy development, including updated scope-of-practice legislation, investment in cold-chain infrastructure, and adaptation of clinical guidelines, must precede a broad rollout. Injectable PrEP offers a transformative opportunity to enhance HIV prevention for those who face the greatest barriers to daily oral adherence; however, realising this potential demands sustained investment in health system strengthening and a research agenda that keeps pace with the rapidly evolving PrEP pipeline.
